# Intensive Care during the COVID-19 Pandemic

**DOI:** 10.3390/vaccines11010125

**Published:** 2023-01-04

**Authors:** Ying Wang, Yuefei Jin, Haiyan Yang

**Affiliations:** Department of Epidemiology, School of Public Health, Zhengzhou University, Zhengzhou 450001, China

## 1. Introduction

The novel coronavirus disease 2019 (COVID-19) pandemic, caused by severe acute respiratory syndrome coronavirus 2 (SARS-CoV-2), has resulted in a sudden sharp rise in hospitalizations for pneumonia with multiorgan disease [1]. COVID-19 manifests with symptoms ranging from fully asymptomatic to severe disease and death, with the most common manifestations being fever, cough, and shortness of breath [2]. Early in infection, the SARS-CoV-2 targets cells, such as bronchial epithelial cells and pneumocytes, through the viral structural spike (S) protein that binds to the angiotensin-converting enzyme 2 (ACE2) receptor [3,4]. Similar to other respiratory viral diseases, including influenza, profound lymphopenia may occur in individuals when the SARS-CoV-2 infects and kills T lymphocyte cells [5]. Moreover, the viral inflammatory response, consisting of both the innate and the adaptive immune responses, also impairs lymphopoiesis and increases lymphocyte apoptosis [5]. In later stages of infection, when viral replication accelerates, the epithelial–endothelial barrier integrity is compromised [5]. In severe cases, fulminant activation of coagulation and consumption of clotting factors occur [6]. Besides, the SARS-CoV-2 infects pulmonary capillary endothelial cells, accentuating the inflammatory response and triggering an influx of monocytes and neutrophils. In addition to the respiratory cells, ACE2 is expressed in many different tissues at different levels; it has an organ-specific distribution, such as in the brain, in the intestinal and vascular smooth muscle cells, and in peripheral organs, including the kidney and the liver [7,8,9]. In light of this fact, the novel coronavirus, together with viral pneumonia, can cause coagulopathy, systemic and local thrombotic events, and kidney and liver injury [10,11].

According to the latest data from the World Health Organization (WHO), the worldwide number of COVID-19 infections have exceeded 651 million, and the total number of deaths have reached 6.65 million [12]. The COVID-19 pandemic has placed an unprecedented demand on modern healthcare resources, particularly with the sudden increase in the need for intensive care for pneumonia with multiorgan disease [13]. The overall in-hospital mortality rate for COVID-19 infections is about 15% to 20%, but as high as 40% of patients require admission to the intensive care unit (ICU) [5]. However, none of the current clinical treatments have alleviated the demand for intensive care. Therefore, priority needs to be given to critical patients and a triage of scarce resources for critical care is needed to preserve the capacity of the ICU for these patients, including mechanical ventilation.

Although many pharmaceutical candidates have been proposed to prevent and treat patients with COVID-19 infections, there is still no effective drug yet. Therefore, in response to the current pandemic, and in addition to non-pharmaceutical interventions (NPIs), such as social distancing, increased hand hygiene, and the wearing of surgical masks, vaccination is expected to be a safe and effective way to help build the immune barrier [10,14,15,16,17,18,19]. To date, there are more than 200 COVID-19 vaccine candidates in various stages of development, and more than 50 vaccine candidates have entered clinical trials, including the use of nucleic acids (DNA or RNA), inactivated or live attenuated viruses, viral vectors, and recombinant proteins or virus particles [20,21,22,23,24,25,26,27,28,29]. However, there is a time lag between vaccine development and distribution in clinics.

We sincerely appreciate all contributors who have submitted their excellent papers to our Special Issue. The current Special Issue provides an effective communication platform to gather original research articles for a more comprehensive understanding of critical care for patients with COVID-19, the challenges posed by the COVID-19 pandemic, and current trends in COVID-19 vaccine development. Three articles have been collected in this Special Issue, mainly covering the care of special populations infected with SARS-CoV-2; antibiotic use and resistance in patients with COVID-19 infections; and COVID-19 vaccines.

## 2. Medical Care for Special Populations with COVID-19

The prone position (PP) helps improve oxygenation through a variety of mechanisms, including increasing the homogeneity of ventilation and perfusion of the entire lung; relieving alveolar compression in the dorsal lung regions; and changing chest compliance, which improves the distribution of gases toward the ventral and para-diaphragmatic lungs. It has been shown to be an effective strategy for patients with severe acute respiratory distress syndrome (ARDS) [30]. Importantly, the impact of PP persists after the patient returns to a supine position. Despite its potential benefits for blood oxygenation, pregnant women are often excluded from trials studying the use of PP due to particular concerns about uterine overload, aortic compression, and fetal health monitoring [31]. Faced with the recent burden of COVID-19 infections among pregnant women, Osmundo et al. described the feasibility, safety, and efficacy of PP in a cohort study that included mechanically ventilated pregnant women who underwent PP due to ARDS induced by the SARS-CoV-2 [32]. Ventilation and gasometric parameters were evaluated at baseline (T0), prone (T1), and supine (T2) positions, and obstetric outcomes were evaluated.

Sixteen pregnant women meeting the inclusion criteria, with a mean age of 31.5 (22.0–46.0) years and a mean body mass index of 36.0 (23.4–47.9) kg/m^2^, participated in their study. Obesity and systemic hypertension were the most common comorbidities, accounting for 73.3% and 40.0% of the cases, respectively. Most of the patients were multiparous (87.5%), and the mean gestational age of the first PP was 27.0 (22.0–31.1) weeks. The study results showed that PP was associated with a >20% increase in PaO_2_ levels and an increase in the PaO_2_/FiO_2_ ratio in 50% and 100% of cases, respectively. Specifically, the PaO_2_/FiO_2_ ratio increased 76.7% (20.5–292.4%) at T1 and 76.9% (0–182.7%) at T2. The authors also found that PP produced sustained improvements in the mean PaO_2_/FiO_2_ ratio (*p* < 0.001) and PaCO_2_ level (*p* = 0.028). An increase greater than 20% in the PaO_2_/FiO_2_ ratio indicates a patient is a responder to PP [33]. Maintaining low levels of PaCO_2_ facilitates gas exchange between the mother and the fetus and is important for avoiding fetal distress during the treatment of a pregnant woman with ARDS. Interestingly, the authors also observed a significant decrease in PaCO_2_ after PP. Remarkably, there were no cases of emergency cesarean section or suspected fetal distress throughout PP or in the first 24 h after returning to the supine position.

This study verifies that PP seems to be effective in improving the PaO_2_/FiO_2_ ratio and PCO_2_ level in pregnant patients with COVID-19 infections, and may help delay preterm deliveries, even after the uterus is enlarged throughout the second and third trimesters.

## 3. Antibiotic Use and Resistance in Patients with COVID-19

According to WHO, antibiotic resistance is one of the top ten global public health hazards to humans [34]. Antimicrobial therapy plays a central role in the management of confirmed or suspected bacterial or fungal respiratory infections. A study before the COVID-19 pandemic predicted that antibiotic-resistant bacteria were responsible for 5 million indirect deaths and 1.3 million direct deaths each year [35]. Unfortunately, those most vulnerable to COVID-19 infection are also most vulnerable to drug-resistant bacteria. A serious observation was reported that approximately 50% of the deceased patients with COVID-19 had bacterial and fungal coinfections which were highly resistant to several antimicrobials [36]. To understand antibiotic use and resistance in patients with COVID-19 in ICU, Kabrah et al. conducted a cross-sectional study of COVID-19 positive patients admitted to an ICU [37].

In this study, the authors performed a drug susceptibility test for isolated strains in 42 COVID-19 positive patients admitted to a hospital’s ICU, following standard protocols. Their study showed that a high percentage (42.7%) of the obtained samples contained *Klebsiella pneumoniae*, and that all the bacteria were multidrug-resistant. Additionally, 76.2% of bacteria were resistant to Ampicillin, 66.7% to Ciprofloxacin, 64.3% to Levofloxacin, 57.1% to Imipenem, and 57.1% to Moxifloxacin. The large rate of multidrug-resistant bacteria (MDRB) acquisition in patients with COVID-19 is alarming. The prevalence of MDRB was assessed in 2020 and compared to the years 2017-2019; according to Aurilio C. et al., the prevalence of overall MDRB infection was 45.2% in 2017, 44.2% in 2018, 41.4% in 2019, 19.2% in 2020 in non-COVID-19 wards, and 29.3% in COVID-19 wards [35]. A further effective defense against MDRB cross-transmission should have been the physical separation of patients with COVID-19. In addition to infection control measures, patients with severe specific infectious pneumonia are first considered to be at significant risk for bacterial co-infection and secondary nosocomial infection; even in the absence of bacterial infection, early COVID-19 symptoms may encourage the initiation of antibiotic treatment [38,39]. Thus, in the event of a pandemic, antimicrobial surveillance is critical to detect warning indicators of abuse or overuse.

Overall, Kabrah et al. showed that antibiotic resistance is highly prevalent among ICU patients with COVID-19 and all of the obtained bacteria were MDRB. Therefore, during the COVID-19 pandemic, it should be emphasized that a multi-modal strategy of strict antibiotic use combined with infection, prevention, and control practices can improve antimicrobial management and, thereby, prevent the spread of MDRB [40].

## 4. Progress on COVID-19 Vaccines

Among the most cost-effective strategies for preventing viral infections, vaccination represents the best tool for helping the immune system to activate protective responses [41]. Vaccination can contribute to preventing or controlling the spread of contagious viral diseases by activating the host immune system to induce long-term immune memory [42]. Several types of vaccines have been developed against the SARS-CoV-2, including inactivated vaccines, live attenuated vaccines, viral vector vaccines, recombinant protein vaccines, and genetic-based vaccines (mRNA and DNA) [41]. Recombinant protein vaccines form the largest proportion of candidate vaccines undergoing clinical trials [43]. The SARS-CoV-2 contains four structural proteins, including spike (S), envelope (E), membrane (M), and nucleocapsid (N) proteins. The S1 subunit of the SARS-CoV-2 spike protein possesses a receptor-binding domain (RBD), which binds to ACE2 of the host cell membrane [44]. The virus releases its genomic RNA into the cytoplasm for reverse transcription and gene expression, resulting in the infection of the host cell. Therefore, the RBD of viral S1 protein is the most targeted antigen in the development of a vaccine against the SARS-CoV-2 [45,46]. The key antigen structure of ZifiVax (ZF2001, Anhui Zhifei Longcom Biopharmaceutical Co., Ltd., Anhui, China) is the R319 to K537 sequence chain in the RBD domain of the SARS-CoV-2 as a key bone, and the two chains are connected by a disulfide bond [47,48]. An efficacy study of ZF2001 has shown that the vaccine can protect mice and rhesus monkeys against SARS-CoV-2 challenge [48]. In order to advance the study of ZF2001 in clinical trials, Yang et al. investigated the general toxicity and immunogenicity of ZF2001 in cynomolgus monkeys and evaluated possible target organs for vaccine toxicity [43].

Yang et al. found that ZF2001 vaccination induced a strong humoral immune response against SARS-CoV-2. They observed high titers and time-dependent neutralizing antibody responses in cynomolgus monkeys. In addition, they observed that increasing the number of vaccinations could increase the antibody level to a certain extent. On the other hand, ZF2001 activated cellular immune responses. High levels of IL-12, IFN-γ, and IL-4 were induced by the stimulation of splenic lymphocyte isolated from cynomolgus monkey with specific COVID-19 antigen, suggesting enhanced Th1 and Th2 immune responses [49]. Therefore, ZF2001 could induce humoral and cellular immune responses in cynomolgus monkeys. These findings suggest that ZF2001 is a promising human vaccine for COVID-19. These results also showed that the ZF2001 vaccine not only did not cause weight/ratio changes in the spleen or thymus organs, but also did not cause histological changes, except for local lymph node hyperplasia and immune response in the muscle at the injection site, which were thought to be caused by the aluminum-containing adjuvant; these symptoms were somewhat relieved after a 2-week recovery period. The antibody-dependent enhancement (ADE) and vaccine enhancement disease (VED) risks caused by SARS-CoV-2 vaccines have been paid more attention to in recent safety evaluations [50,51]. The results of the aforementioned study showed no abnormal changes in immunotoxicity and systemic toxicity after ZF2001 injection in cynomolgus monkeys. As a result, ZF2001 may not increase the risk of ADE and VED in cynomolgus monkeys.

Taken together, Yang et al. demonstrated good tolerance, strong immunogenicity, no general toxicity, and no immunotoxicity of the ZF2001 vaccine, providing support for its entry into large-scale clinical trials.

## 5. Summary and Conclusions

As the COVID-19 pandemic continues to spread, countries around the world are pulling out all the stops to achieve early breakthroughs at all stages of prevention, treatment, and prognosis. Even if SARS-CoV-2 had only infected a small fraction of the planet’s 7.8 billion people, thousands of people became critically ill and required treatment in ICU. The ICU community must prepare for this potentially overwhelming surge of patients to enable rapid diagnosis and isolation, clinical management, and infection prevention. Various problems, such as antibiotic resistance, that arise with the treatment of patients with COVID-19 also need to be addressed in advance. At the same time, current vaccination strategies and preventive measures are the cornerstone of ensuring an optimal protection for all individuals, and research should be strengthened in these areas.
